# Anti-thyroid antibodies and thyroid echo pattern at baseline as risk factors for thyroid dysfunction induced by anti-programmed cell death-1 antibodies: a prospective study

**DOI:** 10.1038/s41416-020-0736-7

**Published:** 2020-02-03

**Authors:** Norio Okada, Shintaro Iwama, Takayuki Okuji, Tomoko Kobayashi, Yoshinori Yasuda, Eri Wada, Takeshi Onoue, Motomitsu Goto, Mariko Sugiyama, Taku Tsunekawa, Hiroshi Takagi, Daisuke Hagiwara, Yoshihiro Ito, Hidetaka Suga, Ryoichi Banno, Tetsunari Hase, Masahiro Morise, Mitsuro Kanda, Kenji Yokota, Naozumi Hashimoto, Masahiko Ando, Yasushi Fujimoto, Masato Nagino, Yasuhiro Kodera, Mitsuhiro Fujishiro, Hideharu Hibi, Michihiko Sone, Hitoshi Kiyoi, Momokazu Gotoh, Yuichi Ando, Masashi Akiyama, Yoshinori Hasegawa, Hiroshi Arima

**Affiliations:** 1 Department of Endocrinology and Diabetes, Nagoya University Graduate School of Medicine, Nagoya, Japan; 2 Department of Respiratory Medicine, Nagoya University Graduate School of Medicine, Nagoya, Japan; 3 Department of Gastroenterological Surgery (Surgery II), Nagoya University Graduate School of Medicine, Nagoya, Japan; 4 Department of Dermatology, Nagoya University Graduate School of Medicine, Nagoya, Japan; 5 Center for Advanced Medicine and Clinical Research, Nagoya University Hospital, Nagoya, Japan; 6 Department of Otorhinolaryngology, Nagoya University Graduate School of Medicine, Nagoya, Japan; 7 Division of Surgical Oncology, Department of Surgery, Nagoya University Graduate School of Medicine, Nagoya, Japan; 8 Department of Gastroenterology and Hepatology, Nagoya University Graduate School of Medicine, Nagoya, Japan; 9 Department of Oral and Maxillofacial Surgery, Nagoya University Graduate School of Medicine, Nagoya, Japan; 10 Department of Hematology and Oncology, Nagoya University Graduate School of Medicine, Nagoya, Japan; 11 Department of Urology, Nagoya University Graduate School of Medicine, Nagoya, Japan; 12 Department of Clinical Oncology and Chemotherapy, Nagoya University Hospital, Nagoya, Japan

**Keywords:** Predictive markers, Autoimmunity

## Abstract

**Background:**

Anti-programmed cell death-1 (PD-1) antibodies can cause thyroid dysfunction. However, no predictive biomarkers enabling stratification of thyroid dysfunction risk have been identified.

**Methods:**

A total of 209 patients treated with an anti-PD-1 antibody were evaluated for anti-thyroid antibodies at baseline and prospectively for thyroid function every 6 weeks for 24 weeks after treatment initiation, and then observed until the visits stopped. Thyroid ultrasonography was performed if the patient was positive for anti-thyroid antibodies at baseline.

**Results:**

Of the 209 patients, 19 (9.1%) developed thyroid dysfunction (destructive thyroiditis or hypothyroidism). The cumulative incidence of thyroid dysfunction was significantly higher in patients who were positive vs. negative for anti-thyroid antibodies (15/44 [34.1%] vs. 4/165 [2.4%], *p* < 0.001). Forty-two patients positive for anti-thyroid antibodies at baseline were divided into two groups according to the presence of an irregular echo pattern. The cumulative incidence of thyroid dysfunction was significantly higher in those with an irregular vs. a regular echo pattern (13/23 [56.5%] vs. 1/19 [5.3%], *p* = 0.001). None of the patients developed thyroid dysfunction after the initial 24-week period.

**Conclusions:**

The risk of thyroid dysfunction induced by anti-PD-1 antibodies can be predicted by evaluation of anti-thyroid antibodies and the thyroid echo pattern at baseline.

**Trial registration:**

UMIN000019024.

## Background

Immune checkpoint inhibitors (ICIs) have been widely used for the treatment of several types of advanced malignancies. ICIs including monoclonal antibodies against cytotoxic T-lymphocyte antigen-4 or programmed death-1 (PD-1) or its ligand PD-L1 exert antitumour effects by eliciting immune reactions against cancer cells. The anti-PD-1 antibodies pembrolizumab and nivolumab have been approved for the treatment of several advanced malignancies, including malignant melanoma (MM)^1,2^ and non-small-cell lung cancer (NSCLC).^3,4^ In addition to their antitumour effects, ICIs have been reported to cause characteristic adverse events, called immune-related adverse events (irAEs),^5^ in the lung, skin, gastrointestinal tract, liver and endocrine glands.

Among the endocrine irAEs, thyroid dysfunction occurs most frequently in patients treated with anti-PD-1 antibodies.^6,7^ Recently, we showed that the cumulative incidence of destructive thyroiditis was significantly higher in patients positive for anti-thyroid antibodies (anti-thyroglobulin antibodies [TgAb] and/or anti-thyroid peroxidase antibodies [TPOAb]) prior to the initiation of nivolumab treatment than in those who were negative for the antibodies (50% vs. 1.7%),^8^ suggesting that the presence of anti-thyroid antibodies at baseline is a predictive biomarker of nivolumab-induced thyroid dysfunction. However, it remains to be clarified whether this is also the case for pembrolizumab, another anti-PD-1 antibody.

Thyroid ultrasonography is commonly used to evaluate autoimmune thyroiditis, and hypoechogenicity and/or irregular echo patterns suggest the presence of inflammatory infiltrates and destruction of the thyroid follicular architecture.^9,10^ It has been reported that thyroid hypoechogenicity is associated with hypothyroidism in Hashimoto thyroiditis,^9,11^ and is a potential biomarker for the development of hypothyroidism.^11,12^ Indeed, irregular echo patterns on thyroid ultrasonography were associated with increased levels of TSH and TPOAb.^13^ Furthermore, previous studies showed that internal echogenicity was lower and irregular after the development of thyroid dysfunction induced by nivolumab.^8,14^ A recent study reported that hypoechogenicity on thyroid ultrasonography was associated with the development of thyroid dysfunction induced by ipilimumab and nivolumab combination therapy.^15^ However, as only one patient had TPOAb at baseline in that study, the significance of hypoechogenicity on thyroid ultrasonography in the rest of the subjects was unclear.^15^

In this study, we performed thyroid ultrasonography in patients with malignancies who were positive for anti-thyroid antibodies before anti-PD-1 antibody treatment. We then examined the potential role of an irregular echo pattern on thyroid ultrasonography as a predictive marker of thyroid dysfunction induced by anti-PD-1 antibodies in these patients.

## Subjects and methods

### Patients

To clarify the clinical features of endocrine irAEs, we initiated a prospective study of irAEs in patients treated with ICIs, including pembrolizumab and nivolumab, on November 2, 2015 (UMIN000019024). All patients (*n* = 209) with MM (*n* = 41), NSCLC (*n* = 91), urothelial cell carcinoma (*n* = 8), renal cell carcinoma (*n* = 21), head and neck cancer (*n* = 28), gastric cancer (*n* = 19) or Hodgkin lymphoma (*n* = 1) treated with pembrolizumab or nivolumab between November 2, 2015 and October 31, 2018 at Nagoya University Hospital were included in this study, and observed until death or referral to another hospital. Patients treated with a combination of immune checkpoint inhibitors were excluded from this study. Written informed consent was obtained from all patients. This study was approved by the Ethical Committee of Nagoya University Hospital. Pembrolizumab (200 mg) was administered to patients every 3 weeks, except to patients with MM, who were treated with 2 mg/kg pembrolizumab every 3 weeks. Nivolumab (3 mg/kg) was administered every 2 weeks, except to patients with MM, who were treated with 2 mg/kg every 3 weeks or 3 mg/kg every 2 weeks. Pembrolizumab or nivolumab treatment continued until disease progression, death, unacceptable severe adverse events or withdrawal of consent. All patients were observed for the development of thyroid dysfunction for at least 24 weeks after treatment initiation. Of the 147 patients treated with nivolumab in this study, 66 were included in our previous study.^8^

### Assessments

To examine thyroid dysfunction, the serum levels of free T3 (FT3), free T4 (FT4), thyroid-stimulating hormone (TSH), thyroglobulin, TgAb, TPOAb and TSH receptor antibodies (TRAb) were assessed at baseline and every 6 weeks for 24 weeks after the first administration of pembrolizumab or nivolumab. After this period, thyroid function was evaluated if clinically indicated. Serum levels of FT3, FT4, TSH, TPOAb, TgAb and TRAb were measured as described previously.^8^

In this study, thyroid dysfunction was monitored and graded by using the Common Terminology Criteria for Adverse Events (CTCAE), version 4.0. Thyroid dysfunction was defined as destructive thyroiditis, hyperthyroidism or hypothyroidism, based on the criteria established by the Japan Thyroid Association. Destructive thyroiditis was defined as a decreased TSH level, elevated FT3 and/or FT4 level and TRAb negativity. Hyperthyroidism was defined as a decreased TSH level, elevated FT3 and/or FT4 level, increased thyroid uptake of ^99m^Tc pertechnetate on scintigraphy and TRAb positivity. Hypothyroidism was defined as an increased TSH level and decreased FT4 level. Ultrasonography of the thyroid glands was performed in patients positive for TgAb and/or TPOAb at baseline, as well as those who developed thyroid dysfunction after the initiation of pembrolizumab or nivolumab. In addition to the assessments conducted every 6 weeks, thyroid function tests were performed as clinically needed during the follow-up period.

**Fig. 1:**
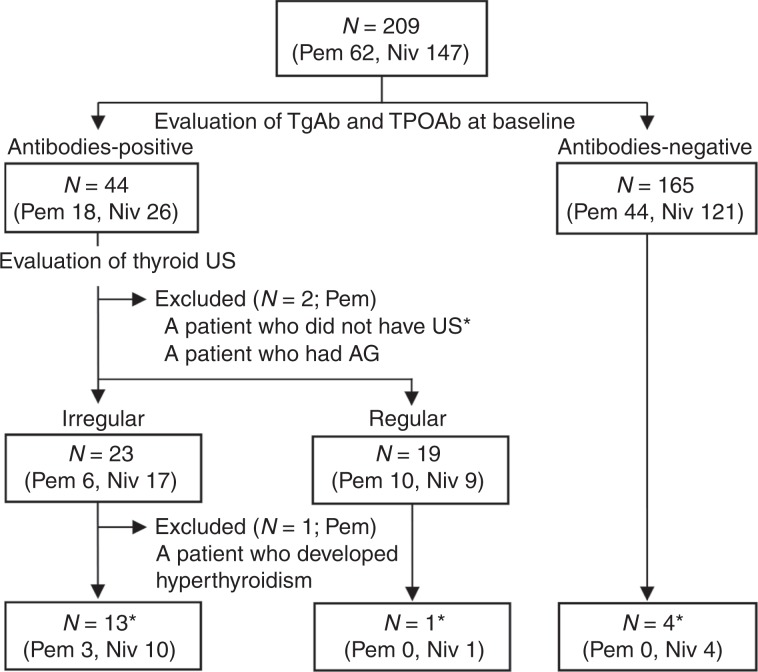
Flow diagram of the patients included in this study. *Patients who developed destructive thyroiditis and/or hypothyroidism induced by anti-PD-1 antibodies. Pem pembrolizumab, Niv nivolumab, US ultrasonography, AG adenomatous goitre, TPOAb anti-thyroid peroxidase antibodies, TgAb anti-thyroglobulin antibodies.

To clarify the significance of ultrasonography findings in the thyroid for predicting the development of thyroid dysfunction induced by anti-PD-1 antibodies, the echo pattern was evaluated by thyroid ultrasonography. In this study, 18 of the 62 patients treated with pembrolizumab and 26 of the 147 treated with nivolumab were positive for anti-thyroid antibodies at baseline (Fig. 1). Among the 44 patients who were positive for anti-thyroid antibodies at baseline, 42 were subsequently evaluated by thyroid ultrasonography, and included in the analysis of the association between an irregular echo pattern and destructive thyroiditis and/or hypothyroidism development. The presence of irregular echo patterns in the thyroid glands was evaluated by the radiologists who performed thyroid ultrasonography, as well as two board-certified endocrinologists (N.O. and S.I.) independently.

### Statistical analysis

Continuous variables (age and follow-up period) are expressed as means ± standard deviation. The significance of differences in continuous variables was assessed using the two-sample *t* test. Nominal variables (tumour type, sex, history of prior treatment with ICIs and positivity for anti-thyroid antibodies at baseline) were compared using Fisher’s exact test. The cumulative incidence of thyroid dysfunction was analysed using the Kaplan–Meier method and compared by log-rank test. All statistical tests were two-sided, and significance was defined as a *p* value < 0.05. All statistical analyses were performed using IBM SPSS Statistics 24 (IBM Corporation, Chicago, IL).

## Results

### Patient characteristics and thyroid dysfunction

A total of 209 patients with malignancies, including MM and NSCLC treated with anti-PD-1 antibodies (62 with pembrolizumab, 147 with nivolumab), were enrolled in this study (Fig. 1, Table 1 and Table 2). After the initiation of pembrolizumab or nivolumab, 5/62 (8.1%) or 15/147 (10.2%) patients, respectively, developed thyroid dysfunction (as defined above) within the 24-week observation period. The thyroid dysfunction grade was < 2 in all patients, except one with grade 3 who developed severe thyrotoxicosis after the initiation of nivolumab, as reported previously.^8^ No thyroid dysfunction was detected after 24 weeks (mean follow-up period: 317 ± 210 days in the pembrolizumab group and 346 ± 362 days in the nivolumab group). Among the patients treated with pembrolizumab, the prevalence of TgAb and/or TPOAb before treatment was significantly higher in the patients who developed thyroid dysfunction compared with those who did not (Table 1). The same pattern was seen among the 147 patients treated with nivolumab (Table 2), consistent with our previous study involving 66 patients treated with nivolumab.^8^ There were no significant differences in the other clinical variables examined.

**Table 1:** Characteristics of the patients treated with pembrolizumab.

	Total	Thyroid dysfunction		*p* value
		(−)	(+)	
	(*n* = 62)	(*n* = 57)	(*n* = 5)	
Tumour type				
MM	17	16	1	0.138
NSCLC	37	33	4	
UC	8	8	0	
Sex				
Male	46	44	2	0.103
Female	16	13	3	
Age, years (range)	69 ± 10	68 ± 10	73 ± 4	0.348
	(42–85)	(42–85)	(68–77)	
Follow-up period (days)	317 ± 210	305 ± 205	444 ± 252	0.159
History of prior immunotherapy	6	6	0	1.000
Positive for anti-thyroid antibodies at baseline	18	13	5	0.001

*MM* malignant melanoma, *NSCLC* non-small-cell lung carcinoma, *UC* urothelial cell carcinoma.

**Table 2:** Characteristics of the patients treated with nivolumab.

	Total	Thyroid dysfunction		*p* value
		(−)	(+)	
	(*n* = 147)	(*n* = 132)	(*n* = 15)	
Tumour type				
MM	24	22	2	0.103
NSCLC	54	50	4	
RCC	21	18	3	
HN	28	24	4	
GC	19	17	2	
HL	1	0	1	
Sex				
Male	104	94	10	0.767
Female	43	38	5	
Age, years (range)	64 ± 12	64 ± 12	63 ± 11	1.000
	(28–86)	(28–86)	(40–78)	
Follow-up period (days)	346 ± 261	340 ± 262	392 ± 255	0.473
History of prior immunotherapy	10	9	1	1.000
Positive for anti-thyroid antibodies at baseline	26	15	11	< 0.001

*MM* malignant melanoma, *NSCLC* non-small-cell lung carcinoma, *RCC* renal cell carcinoma, *HN* head and neck cancer, *GC* gastric cancer, *HL* Hodgkin lymphoma.

The study patients exhibited three different types of thyroid dysfunction. The first was transient thyrotoxicosis without TRAb, observed in 12 patients (2 treated with pembrolizumab, 10 with nivolumab), of whom 9 (2 treated with pembrolizumab, 7 with nivolumab) eventually developed hypothyroidism, suggesting destructive thyroiditis. A representative patient (Pem020) is shown in Supplementary Table S1 and Supplementary Fig. S1A–S1C. The second type of thyroid dysfunction was hypothyroidism without evidence of destructive thyroiditis, observed in seven patients (two treated with pembrolizumab, five with nivolumab). However, as seen in patient Pem010 (Supplementary Table S1 and Supplementary Fig. S1D–S1F), the TSH level was decreased, and FT3 and FT4 levels slightly increased, but within the normal ranges, before the development of hypothyroidism. In addition, this patient showed increased titres of anti-thyroid antibodies and an irregular echo pattern in the thyroid at the onset of hypothyroidism, and eventually required levothyroxine replacement. Among the seven patients who developed hypothyroidism, four (including Pem010) initially experienced a decrease in TSH, but had normal FT3 and FT4 levels. These findings suggest that this type of thyroid dysfunction results from thyroid gland destruction induced by anti-PD-1 antibodies. The third type of thyroid dysfunction was thyrotoxicosis with a positive change in TRAb, observed in patient Pem037 (Supplementary Table S1 and Supplementary Fig. S2A–S2C). Patient Pem037 had a previous history of Graves’ disease, which was in remission at the time of pembrolizumab initiation. The level of ^99m^Tc pertechnetate uptake in the thyroid was increased by 7.1% on scintigraphy (Supplementary Fig. S2D), indicating that the cause of thyrotoxicosis in Pem037 was hyperthyroidism. The levels of TSH, FT3 and FT4 in Pem037 had normalised, and TRAb was no longer detected, at 91 days after the development of hyperthyroidism without requiring medications.

Among the 16 patients who ultimately developed hypothyroidism, 15 required levothyroxine replacement during the observation period, and 1 was referred to another hospital before the initiation of levothyroxine. After the re-administration of pembrolizumab or nivolumab, no patient showed any exacerbation of thyroid dysfunction.

Among the 28 patients with head and neck cancer, 13 received radiotherapy before nivolumab treatment. There was no significant difference in the incidence of thyroid dysfunction between the patients with and those without a history of radiotherapy (2/13 [15.4%] vs. 2/15 [13.3%], *p* = 1.000), suggesting that prior radiotherapy of the thyroid glands has no effect on the development of thyroid dysfunction.

### The presence of anti-thyroid antibodies at baseline is a risk factor for destructive thyroiditis and/or hypothyroidism induced by anti-PD-1 antibodies

**Fig. 2:**
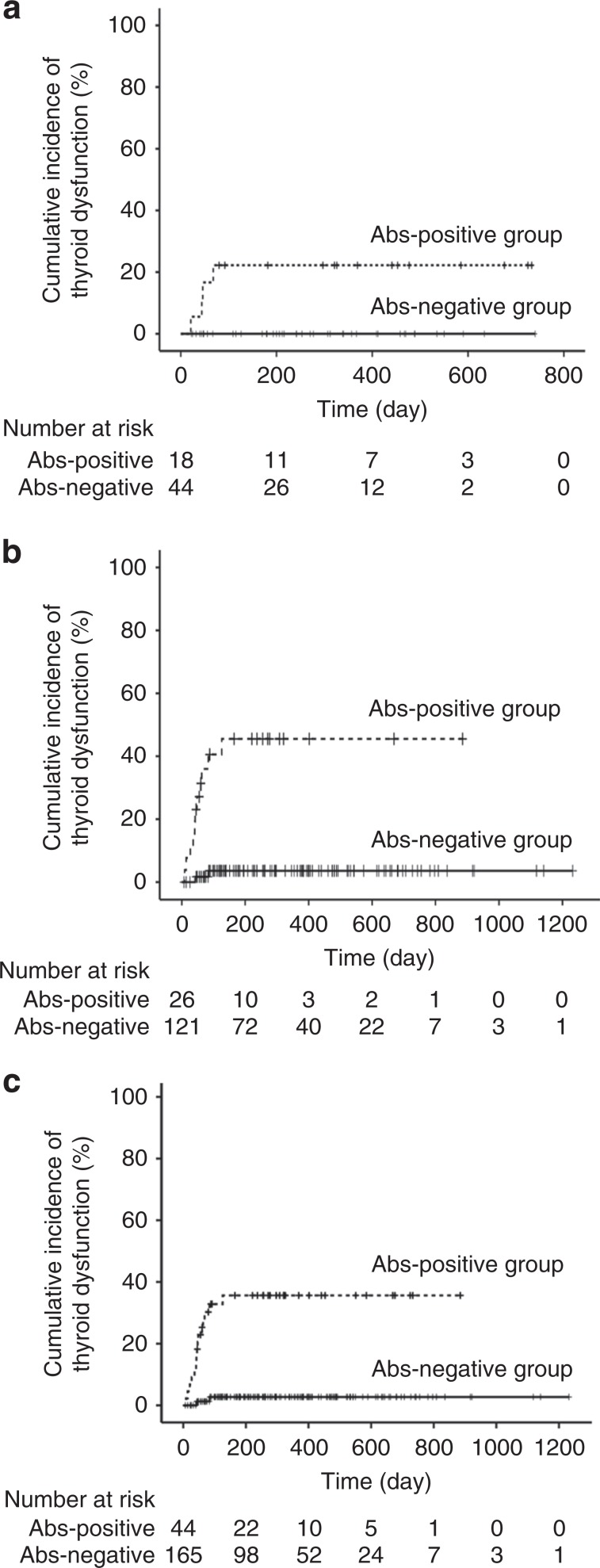
The presence of anti-thyroid antibodies at baseline was associated with the development of destructive thyroiditis and/or hypothyroidism in patients treated with anti-PD-1 antibodies. The cumulative incidence of destructive thyroiditis and/or hypothyroidism was significantly higher in the Abs-positive than Abs-negative group among patients treated with pembrolizumab (**a**), nivolumab (**b**) and pembrolizumab or nivolumab (**c**), respectively. Abs antibodies.

TgAb and/or TPOAb were present at baseline in 44 patients (18 treated with pembrolizumab, 26 with nivolumab) (Abs-positive group); neither antibody was detected in the remaining 165 patients (44 treated with pembrolizumab, 121 with nivolumab) (Abs-negative group) (Fig. 1). During the observation period after treatment initiation, the cumulative incidence of destructive thyroiditis and/or hypothyroidism was significantly higher in the Abs-positive group compared with the Abs-negative group (15/44 [34.1%] vs. 4/165 [2.4%], log-rank test, *p* < 0.001). Among the patients treated with pembrolizumab, the incidence rates of destructive thyroiditis and/or hypothyroidism in the Abs-positive and -negative groups were 4/18 (22.2%) vs. 0/44 (0%) (log-rank test, *p* < 0.01), and among those treated with nivolumab, the respective incidence rates were 11/26 (42.3%) vs. 4/121 (3.3%) (log-rank test, *p* < 0.001) (Fig. 2a–c). These results suggest that the presence of TgAb and/or TPOAb at baseline is a common risk factor for pembrolizumab- as well as nivolumab-induced thyroid dysfunction.

Among the 4 patients who developed thyroid dysfunction in the Abs-negative group, two had a positive anti-thyroid antibody status at thyroid dysfunction onset (positive TgAb and TPOAb in one and positive TgAb in the other patient), suggesting the involvement of thyroid autoimmunity even in patients negative for anti-thyroid antibodies at baseline.

The positive (PPV) and negative predictive values (NPV) of measuring anti-thyroid antibodies at baseline as an indicator of the development of thyroid dysfunction were 15/44 (34.1%) and 161/165 (97.6%), respectively.

### An irregular echo pattern in the thyroid is a further risk factor for destructive thyroiditis and/or hypothyroidism in patients positive for anti-thyroid antibodies at baseline

**Fig. 3:**
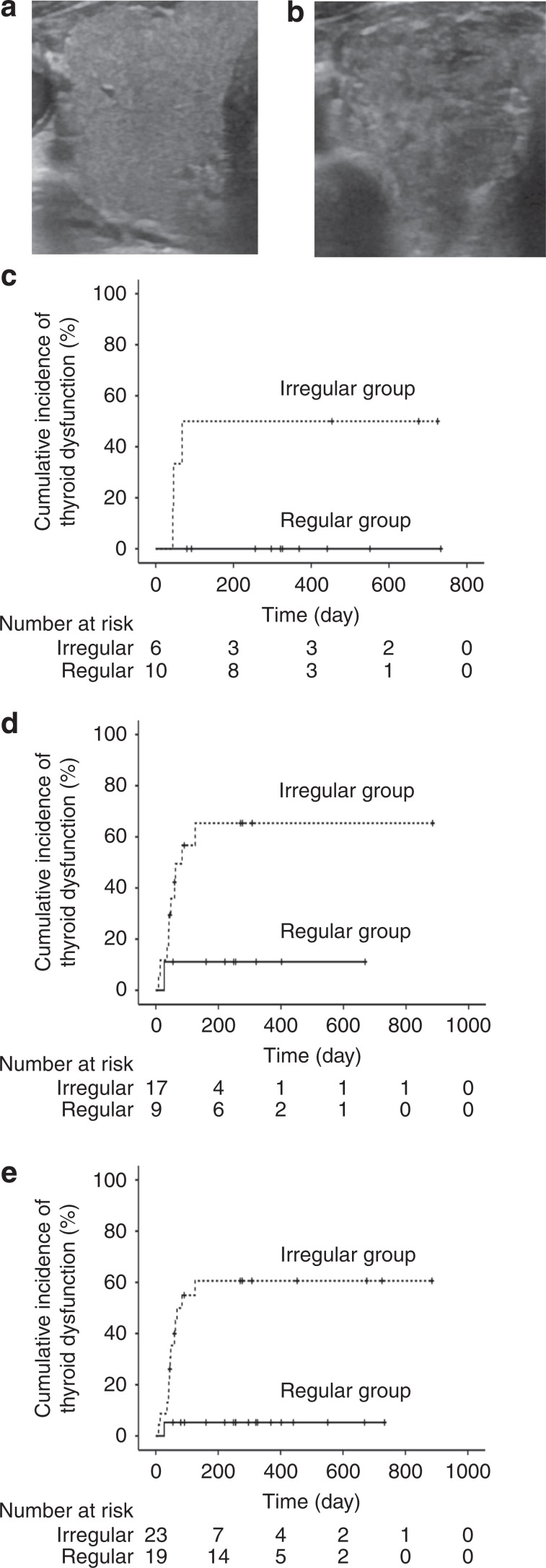
An irregular echo pattern was associated with the development of destructive thyroiditis and/or hypothyroidism in patients treated with anti-PD-1 antibodies. Representative images showing regular (**a**) and irregular (**b**) echo patterns in the thyroid in a patient who was positive for anti-thyroid antibodies at baseline. The cumulative incidence of destructive thyroiditis and/or hypothyroidism was significantly higher in patients with irregular than in those with regular echo patterns among the patients treated with pembrolizumab (**c**), nivolumab (**d**) and pembrolizumab or nivolumab (**e**), respectively.

Among the 44 patients who were positive for anti-thyroid antibodies, 42 were included in the analysis of the association between an irregular echo pattern and destructive thyroiditis and/or hypothyroidism development (Fig. 1). Two patients treated with pembrolizumab were excluded from the analysis for the following reasons: one developed thyroid dysfunction prior to thyroid ultrasonography, and the other could not undergo echo pattern evaluation because of multiple diffuse nodules throughout the entire thyroid gland, possibly due to an adenomatous goitre. In this analysis, the patients were divided into the two groups according to the presence or absence of an irregular echo pattern in the thyroid (Fig. 3a, b). Irregular echo patterns were detected in 23 patients (Irregular group; 6 treated with pembrolizumab, 17 with nivolumab) and regular patterns in the remaining 19 patients (Regular group; 10 treated with pembrolizumab, 9 with nivolumab) (Supplementary Tables S2, S3). The cumulative incidence of destructive thyroiditis and/or hypothyroidism was significantly higher in the patients with irregular vs. regular echo patterns (13/23 [56.5%] vs. 1/19 [5.3%] among those treated with either anti-PD-1 antibody, log-rank test, *p* = 0.001; 3/6 [50%] vs. 0/10 [0%] among those treated with pembrolizumab, log-rank test, *p* < 0.05; 10/17 [58.8%] vs. 1/9 [11.1%] among those treated with nivolumab, log-rank test, *p* < 0.05) (Fig. 3c–e).

The PPV and NPV of evaluating the internal echo pattern in the thyroid gland as an indicator of the development of thyroid dysfunction were 13/23 (56.5%) and 18/19 (94.7%), respectively.

## Discussion

In this prospective study, we analysed the development of thyroid dysfunction in a large number of patients (*n* = 209) treated with anti-PD-1 antibodies, and showed that the presence of anti-thyroid antibodies at baseline is a biomarker of the development of destructive thyroiditis and/or hypothyroidism induced by not only nivolumab but also pembrolizumab. Furthermore, we revealed that among the high-risk patients positive for anti-thyroid antibodies, the presence of an irregular echo pattern in the thyroid at baseline further predicted thyroid dysfunction induced by anti-PD-1 antibodies.

The guidelines for the management of endocrine irAEs, provided by the American Society of Clinical Oncology^16^ and the Society for Endocrinology in the United Kingdom,^17^ recommend testing thyroid function as part of routine clinical monitoring of therapy. The guidelines from the Japan Endocrine Society^18^ introduce our previous finding that anti-thyroid antibody positivity at baseline is a biomarker of thyroid dysfunction induced by nivolumab.^8^ Based on the results of this study, performing thyroid ultrasonography in patients positive for anti-thyroid antibodies at baseline is helpful to identify those patients with a higher risk of thyroid dysfunction.

Thyrotoxicosis induced by anti-PD-1 antibodies is transient, as it results from destruction of the thyroid follicular architecture induced by inflammation.^8,18,19^ However, the natural history of hypothyroidism induced by anti-PD-1 antibodies has not been completely clarified. In this study, nine patients developed hypothyroidism after transient thyrotoxicosis, and seven patients developed hypothyroidism without a prior thyrotoxicosis phase. Among the patients who ultimately developed hypothyroidism and started thyroid hormone replacement therapy, none of them were able to terminate the replacement therapy, indicating that hypothyroidism requiring hormone replacement therapy rarely resolves on its own. It has been reported that thyroid function in patients with painless thyroiditis, a type of destructive thyroiditis seen in patients with Hashimoto thyroiditis, mostly became normal after transient thyrotoxicosis.^20^ These data suggest that the thyroid destruction induced by anti-PD-1 antibodies is much more severe than that seen in painless thyroiditis.

In previous studies, the incidence of thyroid dysfunction induced by pembrolizumab and nivolumab ranged from 5 to 30%,^1–4,21–24^ and the wide range is possibly because the studies were not designed to detect thyroid dysfunction, or the definition of thyroid dysfunction varied among the studies. In this study, we used the definitive diagnostic criteria for destructive thyroiditis, hyperthyroidism and hypothyroidism established by the Japan Thyroid Association. Based on these criteria, the incidence of thyroid dysfunction induced by pembrolizumab was 8.1%, which was similar to that induced by nivolumab (10.2%). The longest duration from the initiation of pembrolizumab or nivolumab to the onset of thyroid dysfunction was 166 or 126 days, respectively. Together with the findings of our previous studies,^8,19^ the greatest susceptibility to developing thyroid dysfunction induced by anti-PD-1 antibodies occurs within the first 6 months after treatment initiation. In a phase 1 clinical trial of pembrolizumab for treatment of NSCLC, Osorio et al.^24^ reported an association between thyroid dysfunction and the presence of anti-thyroid antibodies throughout the follow-up period, although anti-thyroid antibodies at baseline were not examined in all patients. Although other studies reported no association between the TPOAb level and the development of thyroid dysfunction during pembrolizumab treatment,^22,23^ those studies did not evaluate anti-thyroid antibodies in all patients. This study found that the presence of anti-thyroid antibodies at baseline was clinically significant for detecting patients at high risk of thyroid dysfunction induced by nivolumab as well as pembrolizumab. The similar incidence of thyroid dysfunction between the two drugs suggests that thyroid dysfunction is a class effect of anti-PD-1 antibodies.

Several studies have reported that hypoechogenicity or an irregular echo pattern is associated with hypothyroidism,^9,11^ and is predictive of the development of hypothyroidism in patients with Hashimoto thyroiditis.^11,12^ Very recently, a small cohort study (*n* = 28) reported a higher incidence of thyroid dysfunction in patients treated with ipilimumab and nivolumab combination therapy who had widespread hypoechogenicity in the thyroid (5/9 patients) than in those without hypoechogenicity (2/18 patients).^15^ Since that study measured only TPOAb, and not TgAb levels, and included only one patient positive for TPOAb at baseline, the significance of hypoechogenicity in patients positive for anti-thyroid antibodies remains unclear. In this study, we hypothesised that an irregular echo pattern at baseline is a potential biomarker of thyroid dysfunction induced by anti-PD-1 antibodies. We clearly showed that the incidence of destructive thyroiditis and/or hypothyroidism was much higher in patients with an irregular echo pattern on thyroid ultrasonography than in those with a regular pattern. The PPV of measuring anti-thyroid antibodies at baseline as an indicator of the development of thyroid dysfunction was 34.1%. Among the patients positive for anti-thyroid antibodies, the PPV of evaluating the internal echo pattern in the thyroid gland was 56.5%. Therefore, performing thyroid ultrasonography in patients positive for anti-thyroid antibodies is useful for identifying those at higher risk of thyroid dysfunction induced by anti-PD-1 antibodies. The NPV of measuring anti-thyroid antibodies at baseline was 97.6%. Among the patients positive for anti-thyroid antibodies, the NPV of evaluating the internal echo pattern in the thyroid gland was 94.7%. Therefore, these tests can also be useful for identifying patients at low risk of thyroid dysfunction.

Considering the significance of the presence of anti-thyroid antibodies at baseline and an irregular echo pattern, autoimmune reactions against the thyroid glands are likely involved in the pathogenesis of thyroid dysfunction induced by anti-PD-1 antibodies. In a case report, cytotoxic T-cell (CD8^+^) infiltration into the thyroid gland was observed in a patient with nivolumab-induced thyroid dysfunction,^25^ suggesting the involvement of cellular immunity. Considering that hypoechogenicity and/or an irregular echo pattern indicate lymphocyte infiltration,^9,10^ it is strongly suggested that pre-existing lymphocytes infiltrating the thyroid glands are activated by anti-PD-1 antibodies, and are the cause of destructive thyroiditis. In contrast, the role of humoral immunity remains unclear. It was reported that TPOAb induces antibody-dependent cell-mediated cytotoxicity and damage in human thyrocytes.^26,27^ Further studies are needed to clarify the immunological mechanisms of irAEs in the thyroid.

A previous study reported that treatment with anti-PD-1 antibodies can cause a flare-up of a pre-existing autoimmune disease.^28^ In that study, flare-ups occurred in 14/27 (52%) patients with rheumatologic disorders, 3/8 with psoriasis, 2/2 with immune thrombocytopenic purpura and 1/4 with Graves’ disease.^28^ However, it was unclear if hyperthyroidism or destructive thyroiditis was involved in the pathogenesis of thyrotoxicosis in the patient with a Graves’ disease flare-up. Although several case reports have described cases of Graves’ ophthalmopathy induced by ICIs,^29–32^ we describe for the first time a patient (Pem037) who developed hyperthyroidism after pembrolizumab initiation, which was confirmed by increased ^99m^Tc pertechnetate uptake in the thyroid on scintigraphy. Interestingly, the thyrotoxicosis was transient and improved without the need for any anti-thyroid medication. This patient was positive for TPOAb at baseline; however, it is unlikely that the presence of TPOAb at baseline was associated with the development of hyperthyroidism. Therefore, we excluded this patient from the analysis to examine the cumulative incidence of destructive thyroiditis and/or hypothyroidism.

Several studies have reported associations of irAEs, including endocrine irAEs, with improved clinical outcomes.^33–36^ However, it remains unclear which of the endocrine irAEs are predominantly responsible for such outcomes. An association between thyroid dysfunction and longer overall survival was reported in patients with NSCLC treated with pembrolizumab;^24^ thus, it is important to clarify the association between thyroid dysfunction and improved clinical outcomes in not only NSCLC but also other malignancies in the future.

There are some limitations to this study. First, ultrasonography is a subjective measure of the presence of an irregular echo pattern, which is detected by comparing the echogenicity in one area with that in the surrounding areas. The presence of irregular echo patterns in the thyroid was assessed by the radiologists who performed the thyroid ultrasonography, and the two endocrinologists who independently examined all ultrasonography images. The assessment results were the same across all three evaluators in 39 of 42 patients (93%). In the remaining three patients, the consensus of two evaluators was adopted as the final internal echo pattern judgement. Second, the evaluation of thyroid ultrasonography was not performed in patients who were negative for anti-thyroid antibodies at baseline. Given the low incidence of thyroid dysfunction in patients without anti-thyroid antibodies, it is reasonable to perform thyroid ultrasonography exclusively in patients positive for anti-thyroid antibodies, rather than all patients, by starting anti-PD-1 antibody treatment. Third, we excluded one patient who developed pembrolizumab-induced hyperthyroidism from the statistical analyses. Since the pathogenesis of Graves’ disease is completely different from that of destructive thyroiditis, this study provides convincing risk factors for destructive thyroiditis and/or hypothyroidism.

In summary, the risk of thyroid dysfunction induced by anti-PD-1 antibodies is limited within the initial 24 weeks after starting treatment, and can be determined by a two-step process: measurement of anti-thyroid antibodies at baseline followed by evaluation of the echo pattern.

## Supplementary information

supplementary file document

## Data Availability

All data included in this study are available upon request, sent to the corresponding author.
