# Supplementary material for: Anti-thyroid antibodies and thyroid echo pattern at baseline as risk factors for thyroid dysfunction induced by anti-programmed cell death-1 antibodies: a prospective study
Source: Br J Cancer. 2020 Feb 3;122(6):771–7. doi: 10.1038/s41416-020-0736-7 (PMC7078193; doi:10.1038/s41416-020-0736-7)
Supplement: Supplementary file 1 — supplementary file document [file 41416_2020_736_MOESM1_ESM.pdf]

Supplymentary Table S1. Clinical characteristics of representative patients who developed thyroid dysfunction

Case	Age	Sex	Tumor type	TPOAb (IU/ml)		TgAb (IU/ml)		TRAb (IU/ml)		Onset[a] (days)	LT4[c] (µg/day)	Ultrasonography findings at diagnosis
				Pre	Peak	Pre	Peak	Pre	Peak			
Pem020	71	F	MM	38.9[b]	123.5[b]	449.9[b]	850.1[b]	0.3	0.3	21	100	Atrophy, irregular echo pattern
Pem010	77	M	NSCLC	198.6[b]	600[b]	343.2	916.9[b]	0.3	0.6	68	75	Atrophy, irregular echo pattern
Pem037	70	F	NSCLC	176.1[b]	259.4[b]	10	466.3[b]	0.5	2.3[b]	166	Not required	Enlargement, irregular echo pattern

Normal ranges: TPOAb < 16 IU/ml, TgAb < 28 IU/ml, TRAb < 2.0 IU/ml. Abbreviations: TPOAb, anti-thyroid peroxidase antibodies; TgAb, anti-thyroglobulin antibodies; TRAb, anti-thyrotrophin receptor antibodies; LT4, levothyroxine; MM, malignant melanoma; NSCLC, non-small cell lung carcinoma. [a]Days to onset of destructive thyroiditis from the first pembrolizumab treatment. [b]Titer above the upper normal range. [c]The dose of replacement levothyroxine

Supplementary table 2. Characteristics of the patients who were treated with pembrolizumab and evaluated by thyroid ultrasonography

		Total	Irregular echo pattern		*p* value
			(-)	(+)	
		(n = 16)	(n = 10)	(n = 6)	
Tumor type	MM	5	4	1	0.346
	NSCLC	10	5	5	
	UC	1	1	0	
Sex	Male	9	7	2	0.302
	Female	7	3	4	
Age, years (range)		70 ± 6 (58–80)	69 ± 7 (58–80)	71 ± 5 (63–77)	0.609
Follow-up period (days)		430 ± 227	347 ± 197	568 ± 220	0.056

Abbreviations: MM, malignant melanoma; NSCLC, non-small cell lung carcinoma; UC, urothelial cell carcinoma.

Supplementary table 3. Characteristics of the patients who were treated with nivolumab and evaluated by thyroid ultrasonography

		Total	Irregular echo pattern		*p* value
			(-)	(+)	
		(n = 26)	(n = 9)	(n = 17)	
Tumor type	MM	3	2	1	0.302
	NSCLC	8	2	6	
	RCC	3	1	2	
	HN	7	3	4	
	GC	5	1	4	
Sex	Male	16	4	12	0.234
	Female	10	5	5	
Age, years		62 ± 13	62 ± 8	62 ± 15	0.875
(range)		(28–70)	(49–70)	(28–78)	
Follow-up period (days)		326 ± 224	317 ± 188	331 ± 247	0.887

Abbreviations: MM, malignant melanoma; NSCLC, non-small cell lung carcinoma; RCC,

renal cell carcinoma; HN, head and neck cancer; GC, gastric cancer.

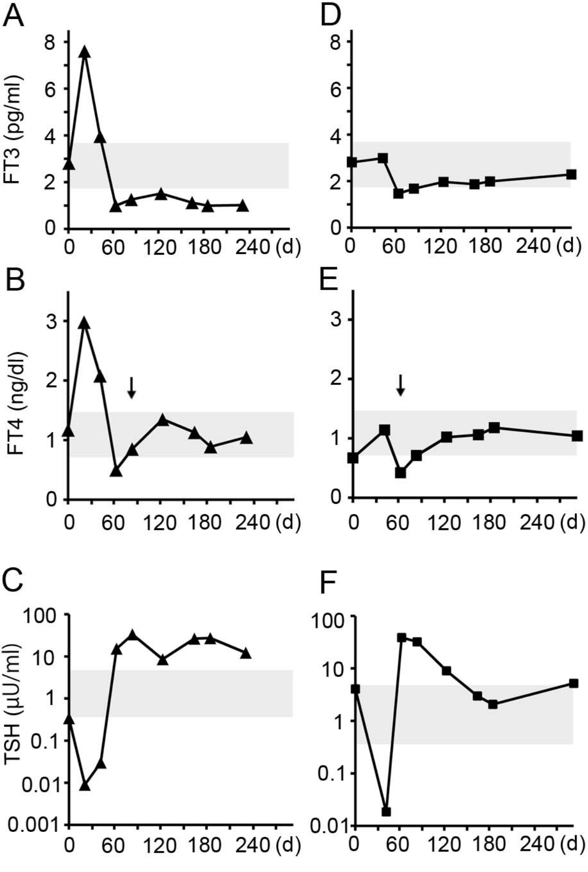

A
FT3 (pg/ml)
0
2
4
6
8
0
60
120
180
240
(d)
B
FT4 (ng/dl)
0
1
2
3
0
60
120
180
240
(d)
C
TSH (µU/ml)
0.001
0.01
0.1
1
10
100
0
60
120
180
240
(d)
D
0
2
4
6
8
0
60
120
180
240
(d)
E
0
1
2
3
0
60
120
180
240
(d)
F
0.01
0.1
1
10
100
0
60
120
180
240
(d)

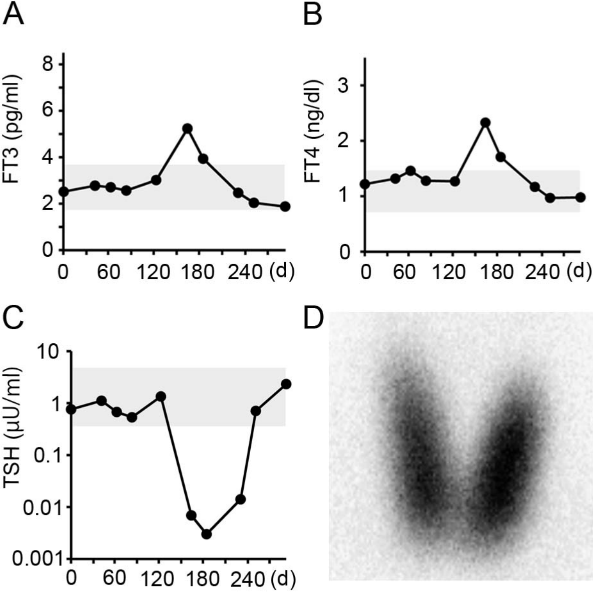
A
FT3 (pg/ml)
0
2
4
6
8
0
60
120
180
240
(d)
B
FT4 (ng/dl)
0
1
2
3
0
60
120
180
240
(d)
C
TSH (μU/ml)
10
1
0.1
0.01
0.001
0
60
120
180
240
(d)
D

Supplementary Figure legends

Supplementary Figure S1. Clinical features of thyroid dysfunction in patients treated with pembrolizumab

Changes in FT3, FT4, and TSH levels over 240 days in a patient who developed destructive thyroiditis (Pem020) (A–C) or hypothyroidism (Pem010) (D–F). Gray areas indicate the normal range of each value. Arrows indicate the day of starting levothyroxine replacement treatment. FT3, serum free triiodothyronine; FT4, serum free thyroxine; TSH, serum thyroid stimulating hormone; (d), day.

Supplementary Figure S2. Clinical features of hyperthyroidism in the patient treated with pembrolizumab

Changes in FT3, FT4, and TSH levels over 240 days in the patient who developed hyperthyroidism (Pem037) (A–C). Gray areas indicate the normal range of each value. A representative image showing the increased level of ^{99m}Tc pertechnetate uptake in the thyroid at the time of thyrotoxicosis diagnosis (D). FT3, serum free triiodothyronine; FT4, serum free thyroxine; TSH, serum thyroid stimulating hormone; (d), day.
